# Prior Antithrombotic Therapy is Associated with Increased Risk of Death in Patients with Intracerebral Hemorrhage: Findings from the Chinese Stroke Center Alliance (CSCA) Study

**DOI:** 10.14336/AD.2020.1205

**Published:** 2021-08-01

**Authors:** Dacheng Liu, Hongqiu Gu, Yuehua Pu, Jingyi Liu, Kaixuan Yang, Wanying Duan, Xin Liu, Ximing Nie, Zhe Zhang, Chunjuan Wang, Xingquan Zhao, Yilong Wang, Zixiao Li, Liping Liu

**Affiliations:** ^1^Department of Neurology, Beijing Tiantan Hospital, Capital Medical University, Beijing, China.; ^2^China National Clinical Research Center for Neurological Diseases, Beijing, China..

**Keywords:** antithrombotic therapy, intracerebral hemorrhage, mortality

## Abstract

The association of preceding antithrombotic therapy with outcomes of patients with intracerebral hemorrhage (ICH) has not been well clarified. We investigated the characteristics and associations of prior antithrombotic therapy (oral anticoagulants, antiplatelet therapy or both) in outcomes of in-hospital patients with ICH. Data were derived from the Chinese Stroke Center Alliance (CSCA) database. Enrolled patients were categorized by the different types of preceding antithrombotic therapy: antiplatelet therapy (APT), oral coagulants (OAs), both OAs and APT use and no-antithrombotic therapy (no-ATT). Among 85705 patients enrolled, 4969 (5.8%), 720 (0.8%), 905 (1.1%) and 79111 (92.3%) patients were on APT, OAs, both OAs and APT, and non-ATT respectively prior to their admission. Crude in-hospital death was 149(3.0%), 41(5.7%), 46(5.1%) and 1781(2.3%) in APT, OAs, both OAs and APT, and non-ATT groups, respectively (P<0.0001). Multivariate analysis revealed that patients in prior OAs (adjusted odds ratio [aOR], 1.95; 95% confidence interval [CI], 1.18-3.21; P=0.0091) and both OAs and APT groups (aOR 1.92, 95% CI 1.17-3.15, P=0.0094) were associated with an increased risk of in-hospital mortality compared with the non-ATT group, but not in those who were on APT (aOR 1.12, 95% 0.93-1.36, P=0.2372). In the subgroup analysis, a stronger association between prior OAs and in-hospital death was found among patients who were older ≥ 65 years (P for interaction is 0.0382). In this nationwide prospective study, prior OAs and concomitant use of OAs and APT but not prior ATP were associated with increased odds of in-hospital mortality compared with ICH patients who were on no-ATT.

Antithrombotic therapy (ATT) including antiplatelet therapy (APT), anticoagulation therapy or a combination of both have been the mainstay therapy for the prevention of embolic vascular events [1, 2]. With the rapid introduction of direct oral anticoagulants (OAs) and combination use with APT, concerns of bleeding complications from prior ATT have been raised. ATT is not only linked with increased risk of bleeding, such as intracerebral hemorrhage (ICH) [3], but also the more severe type of ICH [4-6].

Previous studies have investigated the association of preceding use of OAs or APT with the outcomes of ICH. However, the results were inconsistent due to small sample sizes [7-10]. Limited data were available regarding the characteristics of ICH, and most of the studies focused on the comparison between the patients on single antithrombotic therapy with those on no-ATT. Comparison between OAs, APT and concomitant use of both ATT has not been well studied [11, 12]. By using data from the prospective Chinese Stroke Center Alliance (CSCA) registry, we evaluated the clinical profiles of patients with acute ICH who received prior antithrombotic therapy, and their in-hospital outcomes.

## MATERIALS AND METHODS

### Date Source

The current data are from the CSCA registry, which is a national, hospital-based, multicenter, multifaceted intervention and continuous quality improvement initiative for acute stroke and transient ischemic attack. The details of rationale, design, data collection and management of CSCA have been described earlier[13]. Trained researchers at each participating hospital used a web-based management system (Gaide, Inc., Beijing, China) to collect patient’s clinical data. Participating hospitals had the approval from local Institutional Review Board to collect data in CSCA without requiring individual informed consent under the common rule or a waiver of authorization and exemption since this was a process improvement project. The data were centralized and analyzed by The China National Clinical Research Center for Neurological Diseases.

### Study Population

**Figure 1.**
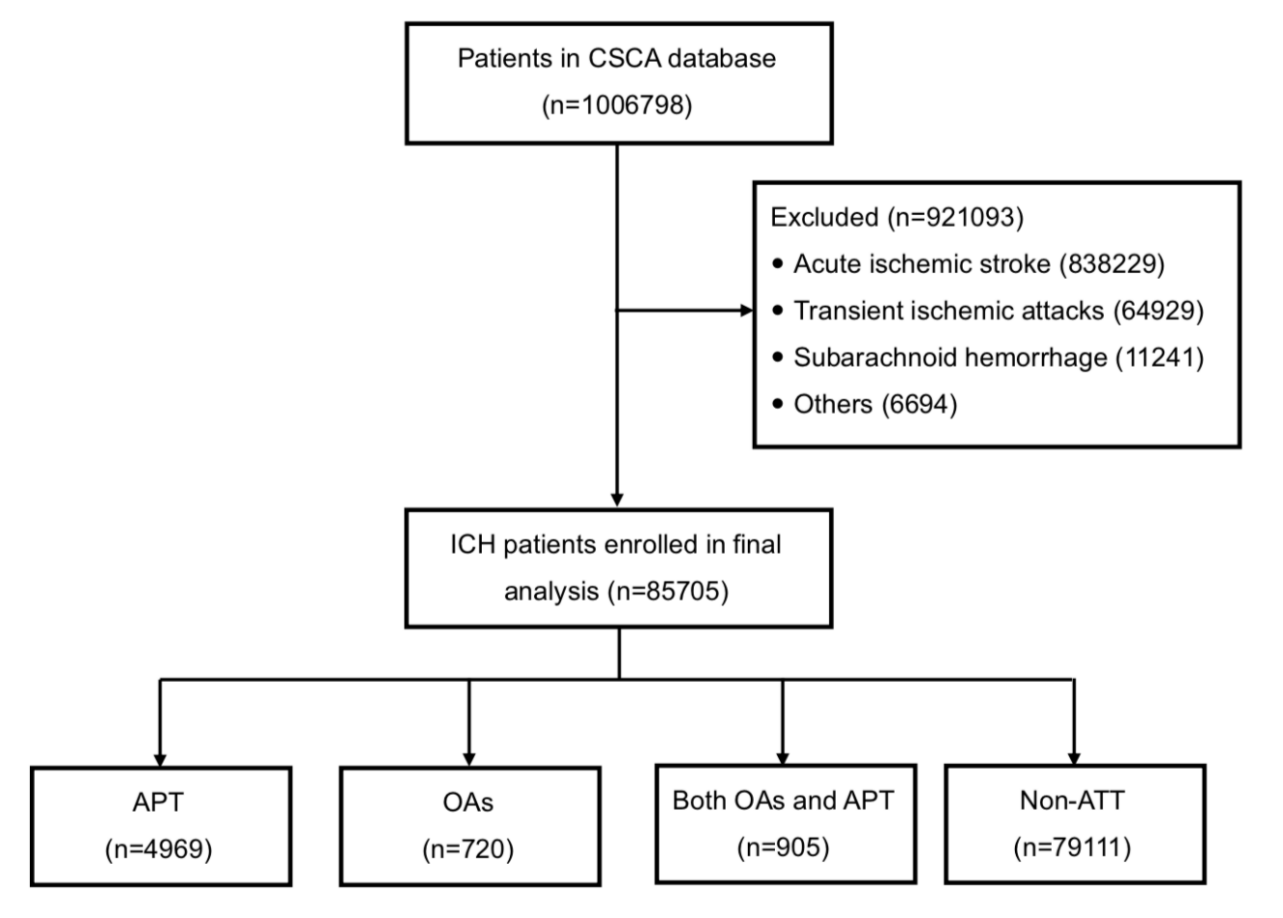
Flow chart of study. CSCA indicates Chinese Stroke Center Alliance; ICH, intracerebral hemorrhage; APT, antiplatelet therapy; OAs, oral anticoagulants; non-ATT, non-antithrombotic therapy.

Patients were identified in CSCA database from August 1, 2015 to July 31, 2019. They were excluded if they were not diagnosed with an ICH. All diagnoses were confirmed by computed tomography scan. Preceding antithrombotic therapy was defined as any use of antiplatelet therapy or oral anticoagulation agents within the 6 months before stroke onset and the antithrombotic therapy must be taken consistently for at least 14 days. Antiplatelet drugs included aspirin, clopidogrel, dipyridamole, ticagrelor, cilostazol or other agents. Oral anticoagulation included vitamin K antagonist (VKA) such as warfarin or novel oral anticoagulation (NOAC) including dabigatran, rivaroxaban, apixaban, etc. All participants were categorized into 4 groups according to their preceding antithrombotic therapy: (1) APT; (2) OAs; (3) Both OAs and APT; (4) non-ATT (Fig. 1).

**Table 1:** Baseline characteristics of study population according to prior anti-thrombotic therapies.

Variables	Total (n=85705)	APT (n=4969)	Both OAs and APT (n=905)	OAs (n=720)	Non-ATT (n=79111)	P value
Age, median (IQR), y	63.0 (53.0-72.0)	67.0 (59.0-75.0)	66.0 (56.0-75.0)	65.0 (55.5-75.0)	63.0 (53.0-72.0)	<0.0001
Male, n, (%)	53543 (62.5)	3231 (65.0)	567 (62.7)	449 (62.4)	49296 (62.3)	0.0021
Medical history, n (%)						
Hypertension	61128 (71.3)	4223 (85.0)	736 (81.3)	533 (74.0)	55636 (70.3)	<0.0001
Diabetes mellitus	8146 (9.5)	965 (19.4)	166 (18.3)	122 (16.9)	6893 (8.7)	<0.0001
Dyslipidemia	3644 (4.3)	522 (10.5)	138 (15.2)	52 (7.2)	2932 (3.7)	<0.0001
Atrial fibrillation	1312 (1.5)	217 (4.4)	79 (8.7)	202 (28.1)	814 (1.0)	<0.0001
Previous stroke	24622 (28.7)	3428 (69.0)	597 (66.0)	328 (45.6)	20269 (25.6)	<0.0001
Prior CHD/MI	4818 (5.6)	844 (17.0)	225 (24.9)	145 (20.1)	3604 (4.6)	<0.0001
Smoking	27865 (32.5)	1807 (36.4)	291 (32.2)	218 (30.3)	25549 (32.3)	<0.0001
Alcohol	20908 (24.4)	1139 (22.9)	245 (27.1)	149 (20.7)	19375 (24.5)	0.0017
Baseline NIHSS score, median (IQR)	6 (2-12)	5 (2-11)	8 (4-16)	8 (3-18)	6 (2 -12)	<0.0001
Baseline NIHSS≥16, n (%)	9514 (11.1)	612 (12.3)	126 (13.9)	121 (16.8)	8655 (10.9)	<0.0001
Baseline GCS score, median (IQR)	13 (8-15)	14 (8-15)	10 (6-14)	12 (7-15)	13 (8-15)	<0.0001
Admission SBP, mmHg, mean ± SD	164.6 ± 28.2	161.3 ± 26.6	162.6 ± 30.1	155.2 ± 26.3	164.9 ± 28.3	<0.0001
Admission DBP, mmHg, mean ± SD	95.3 ± 16.9	92.5 ± 15.8	94.4 ± 17.0	91.2 ± 16.4	95.5 ± 16.9	<0.0001
Biochemical indexes, median (IQR)						
LDL-C, mmol/L	2.6 (2.0-3.2)	2.4 (1.8-3.1)	2.6 (2.0-3.5)	2.5 (1.8-3.5)	2.6 (2.0-3.2)	<0.0001
FBG, mmol/L	5.9 (5.1-7.1)	6.0 (5.1-7.4)	6.0 (5.2-7.5)	6.1 (5.2-7.4)	5.9 (5.1-7.1)	<0.0001
HCY, µmol/L	13.0 (9.5-18.1)	14.0 (10.2-19.3)	13.2 (8.9-20.0)	12.4 (8.6-19.4)	13.0 (9.5-18.0)	<0.0001
Creatinine, µmol/L	67.7 (55.0-85.0)	68.1 (56.0-84.0)	68.0 (54.0-96.7)	73.0 (55.0-97.7)	67.4 (54.8-84.6)	<0.0001
INR	1.0 (0.9-1.1)	1.0 (1.0-1.1)	1.1 (1.0-1.7)	1.2 (1.0-2.6)	1.0 (0.9-1.1)	<0.0001
PLT, 10^9^/L	199 (155-244)	200 (158-245)	196 (151-240)	187 (144-239)	199 (155-244)	0.0002
Pre-admission medications, n (%)						
Antihypertensive	40444 (47.2)	3832 (77.1)	727 (80.3)	474 (65.8)	35411 (44.8)	<0.0001
Cholesterol-lowering	4949 (5.8)	2210 (44.5)	449 (49.6)	158 (21.9)	2132 (2.7)	<0.0001
Antidiabetic	5956 (6.9)	851 (17.1)	153 (16.9)	103 (14.3)	4849 (6.1)	<0.0001
Hospital levels, n (%)						<0.0001
Secondary hospitals	35689 (41.6)	2312 (46.5)	415 (45.9)	192 (26.7)	32770 (41.4)	
Tertiary hospitals	50016 (58.4)	2657 (53.5)	490 (54.1)	528 (73.3)	46341 (58.6)	

APT indicates antiplatelet therapy; OAs, oral anticoagulants; ATT, antithrombotic therapy; IQR, interquartile range; CHD, coronary heart disease; MI, myocardial infarction; NIHSS, National Institutes of Health Stroke Scale; GCS, Glasgow scale; SBP, systolic blood pressure; DBP, diastolic blood pressure; SD, standard deviation; LDL-C, low-density lipoprotein cholesterol; FBG, fasting blood-glucose; HCY, homocysteine; INR, international normalized ratio; and PLT, platelets.

### Outcomes

Primary outcome was in-hospital mortality, defined as all-cause mortality during hospitalization. Secondary outcomes comprised of (1) percentage of hematoma evacuation, including both craniotomy or aspiration; (2) length of hospital stay; (3) in-hospital complications, including pneumonia, dysphagia, pulmonary embolism, post-stroke epilepsy, urinary infection and gastrointestinal bleeding.

### Statistical Analysis

Baseline characteristics were compared across 4 preceding antithrombotic treatment groups. Categorical variables were described as numbers (proportions), and continuous variables as medians (interquartile range, IQR) or means ± SD. χ2 tests were used for univariate analyses of categorical variables. Continuous variables were analyzed by Kruskal-Wallis tests.

Multivariable logistic regression models were performed to assess the relationship between in-hospital mortality and prior ATT use, with no-ATT group as reference. Unadjusted odds ratios (ORs) were first calculated, then three models adjusting potential confounders were used in multivariable logistic regression to evaluate the association between outcomes and preceding ATT use. Model 1 contained confounders as age and sex. Model 2 contained age, sex, medical history (history of hypertension, diabetes mellitus, dyslipidemia, atrial fibrillation, previous stroke, coronary heart disease, smoking, alcohol use), pre-admission medications (antihypertensive therapy, cholesterol-lowing therapy and antidiabetic therapy) and admission blood pressure. Model 3 was performed as sensitivity analysis using the National Institute of Health Stroke Scale (NIHSS) score as an additional covariate. The NIHSS score was not included in the main analysis since higher NIHSS score might be a result of hematoma expansion caused by OAs [14].

Meanwhile, sensitivity analysis was also performed and stratified by clinically relevant subgroups. Relevant stratified groups included age (<65 vs ≥65 years old), sex (female vs male), history of hypertension (yes vs no) and hospital levels (tertiary hospitals vs secondary hospitals), for these variables were important in reference to the pathogeny of ICH and decision process of antithrombotic treatment.

All analyses were performed with SAS 9.4 (SAS Institute Inc, Cary, NC). Two-sided P < 0.05 was considered statistically significant. To enhance the speed and efficiency of report creation and presentation, a SAS macro called %ggBaseline was used to analyze and report baseline results automatically [15].

## RESULTS

### Characteristics of Study Population

A total of 1006798 patients from 1476 participating hospitals were admitted in CSCA database. Among them, 921093 patients were excluded for they were diagnosed as AIS (n=838229), TIA (n=64929), SAH (n=11241) and others (n=6694). A total of 85705 ICH patients were identified and entered into the final analysis without any missing data. Among them, 4969 (5.8%) patients were classified into the APT group, 720 (0.8%) to the OAs group, 905 (1.1%) to both OAs and APT therapy group, and 79111 (92.3%) to the no-ATT group (Fig. 1). Details of the baseline characteristics of total patients and 4 preceding ATT groups were shown in Table 1. In general, median (IQR) age was 63.0 (53.0-72.0); 39205 (45.7%) patients were 65 years or older; 53543 (62.5%) were male and 50016 (58.4%) were treated in tertiary hospitals. All baseline variables were statistically different among these four groups. Patients in APT group had highest percentage of traditional cerebrovascular diseases risk factors such as hypertension (85.0%), diabetes mellitus (19.4%), previous stroke (69.0%) and smoking (36.4%), as compared with other groups. Patients in OAs group had highest percentage of history of atrial fibrillation (28.1%) and highest creatinine level [73.0 (55.0-97.7) µmol/L]. Patients in combined OAs and APT group had highest proportion of coronary heart disease (CHD) and myocardial infarction (MI) (24.9%), and lowest baseline GCS score [10 (6-14)]. Meanwhile, the percentage of the baseline NIHSS≥16 was highest in the OAs group (16.8%), followed by combination therapy group (13.9%), APT group (12.3%) and non-ATT group (10.9%, p<0.0001).

**Table 2:** Primary and secondary outcomes by prior antithrombotic treatment.

Variables	Total (n=85705)	APT (n=4969)	OAs (n=720)	Both OAs and APT (n=905)	Non-ATT (n=79111)	P value
Primary outcome						
In-hospital mortality, n (%)	2017 (2.4)	149 (3.0)	41 (5.7)	46 (5.1)	1781 (2.3)	<0.0001
Secondary outcomes						
Hematoma evacuation, n (%)	8923 (10.9)	377 (8.1)	98 (14.3)	155 (18.7)	8293 (11.0)	<0.0001
Length of hospital stay, median (IQR)	15 (10-21)	15 (10-21)	15 (10-22)	15 (9-23)	15 (10-21)	0.1272
In-hospital complications, n (%)						
Pneumonia	21874 (25.6)	1296 (26.2)	229 (31.9)	254 (28.1)	20095 (25.4)	0.0002
Dysphagia	13785 (16.1)	870 (17.5)	118 (16.4)	179 (19.8)	12618 (15.9)	0.0013
Pulmonary embolism	233 (0.3)	15 (0.3)	8 (1.1)	10 (1.1)	200 (0.3)	<0.0001
Post-stroke epilepsy	1203 (1.4)	92 (1.9)	20 (2.8)	15 (1.7)	1076 (1.4)	0.0003
Urinary infection	2112 (2.5)	164 (3.3)	17 (2.4)	23 (2.5)	1908 (2.4)	0.0014
Gastrointestinal bleeding	2458 (2.9)	185 (3.7)	29 (4.0)	26 (2.9)	2218 (2.8)	0.0005

APT indicates antiplatelet therapy; OAs, oral anticoagulants; ATT, antithrombotic therapy; IQR, interquartile range.

**Table 3:** Associations between in-hospital mortality and prior anti-thrombotic therapies.

Indexes	Unadjusted OR (95% CI)	P value	Model 1* Adjusted OR (95% CI)	P value	Model 2 † Adjusted OR (95% CI)	P value	Model 3 ‡ Adjusted OR (95% CI)	P value
APT	1.34 (1.06, 1.71)	0.0151	1.23 (0.98, 1.55)	0.0807	1.12 (0.93, 1.36)	0.2372	1.24 (0.82, 1.87)	0.3154
OAs	2.63 (1.79, 3.85)	<0.0001	2.50 (1.73, 3.63)	<0.0001	1.95 (1.18, 3.21)	0.0091	1.30 (0.54, 3.10)	0.5554
Both OAs and APT	2.34 (1.52, 3.63)	0.0001	2.21 (1.42, 3.44)	0.0004	1.92 (1.17, 3.15)	0.0094	0.55 (0.24, 1.28)	0.1675
Non-ATT	Reference		Reference		Reference		Reference	

OR indicates odds ratio; CI, confidential interval; APT, antiplatelet therapy; OAs, oral anticoagulants; ATT, anti-thrombotic therapy, NA, not available; TIA, transient ischemic attack; CHD, coronary heart disease; MI, myocardial infarction, SBP, systolic blood pressure and DBP, diastolic blood pressure, NIHSS, National Institute of Health Stroke Scale. * Adjusted for age, sex † Adjusted for age, sex, medical history (hypertension, diabetes mellitus, dyslipidemia, atrial fibrillation, previous stroke/TIA, prior CHD/MI, smoke, alcohol), pre-admission medication, SBP, DBP. ‡ Adding NIHSS in sensitivity analysis, adjusted for age, sex, Medical History (hypertension, diabetes mellitus, dyslipidemia, atrial fibrillation, previous stroke/TIA, Prior CHD/MI, history of smoke, history of alcohol), Pre-admission medication, SBP, DBP, and NIHSS score.

### Preceding Antithrombotic Treatment and Outcomes

Crude in-hospital mortality was 3.0% (149/4969), 5.7% (41/720), 5.1% (46/906), 2.3% (1781/79111) in APT groups, OAs group, both OAs and APT group, and non-ATT group, respectively (p<0.0001, Table 2). About 18.7% of patients in combination therapy group and 14.3% of patients in OAs group received hematoma evacuation treatment, in contrast to 8.1% of patients in APT group and 11.0% of patients in non-ATT group (p<0.0001). The duration of hospital stays had no difference between 4 groups (p=0.1272). As for in-hospital complications, patients in the OAs group had the highest proportion of pneumonia (31.9%), post-stroke epilepsy (2.8%) and gastrointestinal bleeding (4.0%), (all p <0.0001), while patients with combined OAs and APT had the highest percentage of dysphagia (19.8%, p=0.0013).

In unadjusted analysis, compared with no preceding antithrombotic therapy, prior APT use (OR 1.34, 95% confidence interval [CI] 1.06-1.71), prior anticoagulation use (OR 2.63, 95% CI 1.79-3.85) and concomitant prior use of OAs and APT (OR 2.34, 95% CI 1.52-3.63) were associated with an increased odds ratio of in-hospital mortality. In model 1 analysis after adjusting for age and sex, such trend remained in groups of prior OAs (adjusted OR [aOR], 2.50; 95% CI 1.73-3.63. p<.0001) and concomitant use of OAs and APT (aOR 2.21, 95% CI 1.42-3.44. p=0.0004). In model 2 after adjusting for potential clinical covariables, patients in groups of prior OAs use (aOR 1.95, 95% CI 1.18-3.21. p=0.0091) and concomitant use of OAs and APT (aOR 1.92, 95% CI 1.17-3.15. p=0.0094) still had significantly increased risk of in-hospital mortality compared with those on no-ATT, but not seen as compared to patients on APT (aOR 1.12, 95% CI 0.93-1.36. p=0.2372) (Table 3). However, in sensitivity analysis, after adjusting for the NIHSS score, a non-significant trend of higher in-hospital death rate was found in prior APT, OAs and both groups compared with non-APT group (Table 3).

### Subgroup Analysis

**Figure 2.**
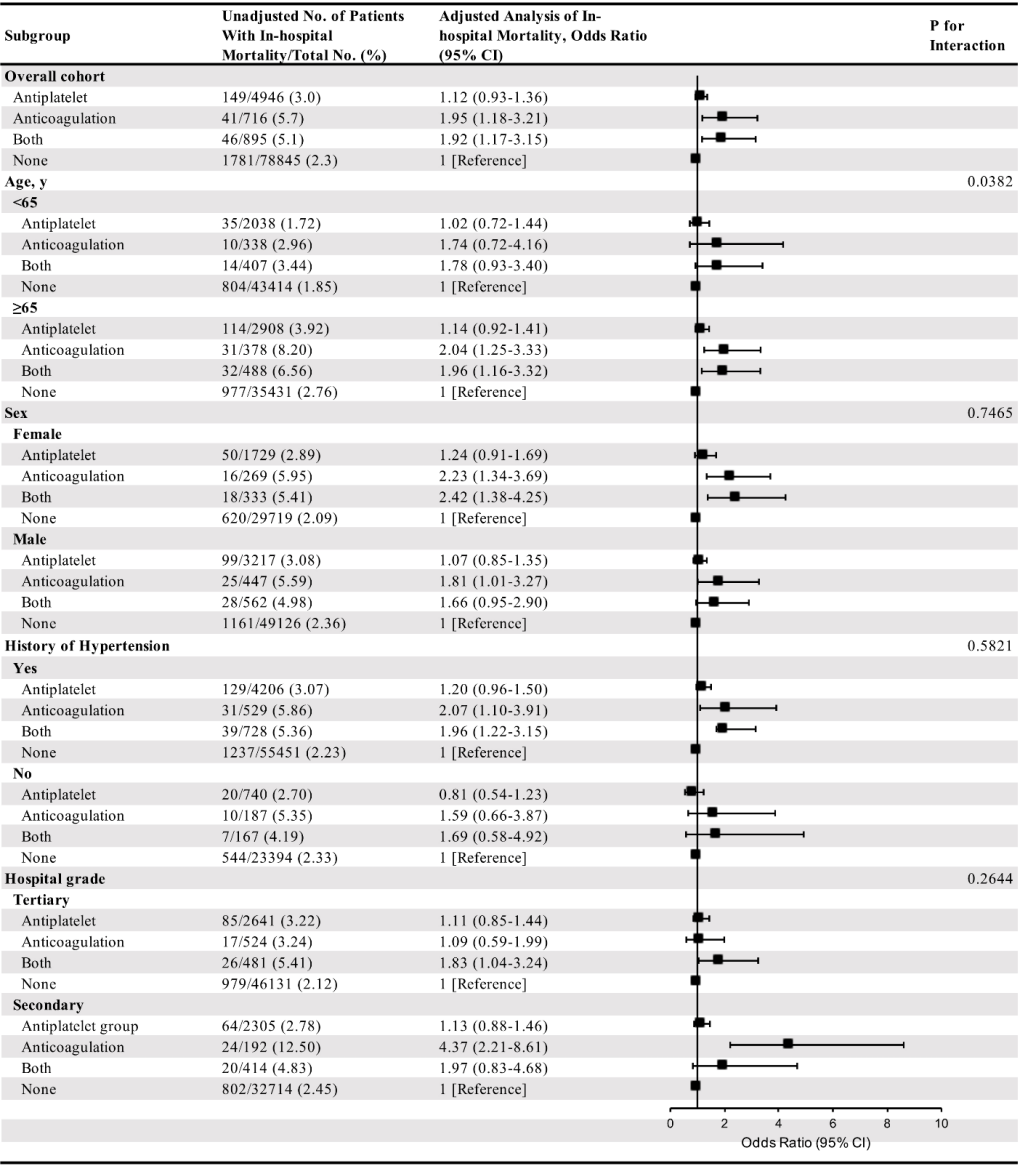
Subgroup analysis about association between preceding antithrombotic treatment and in-hospital mortality. CI indicates confidence interval.

In further subgroup analysis, stratified by age, a significant interaction was found between prior antithrombotic therapy and in-hospital mortality (P for interaction is 0.0382, Fig. 2). Prior OAs or both OAs and APT groups (aOR 2.04, 95% CI 1.25-3.33; aOR 1.96, 95% CI 1.16-3.32; respectively) were associated with in-hospital mortality in patients with age ≥65 years old rather than in patients with age < 65 years old (aOR 1.74, 95% CI 0.72-4.16; aOR 1.78, 95% CI 0.93-3.40; respectively) compared with non-ATT group. As for other subgroups, no significant interaction was observed between prior antithrombotic therapy and sex, history of hypertension or hospital grades, respectively (Fig. 2).

## DISCUSSION

In this large, nationwide multicenter registry study, we found that preceding anticoagulation therapy and concomitant use of anticoagulation and antiplatelet therapy, but not prior antiplatelet therapy alone, were associated with increased odds of in-hospital mortality compared with non-antithrombotic therapy in patients admitted for ICH. In stratified subgroup analysis, higher in-hospital mortality with prior OAs and concomitant use of OAs and APT were found in patients older than 65.

In univariable and multivariable analysis, patients with preceding APT, both OAs and OAs and APT use were all associated with higher in-hospital mortality compared with those on no-ATT. However, after adjusting for age and sex, only both OAs and APT group and OAs group had higher mortality than the reference group. With the increased numbers of adjusting confounders in model 2, patients in both the OAs and APT groups and preceding OAs group were still associated with higher in-hospital mortality compared with those on no-ATT. However, the adjusted odds ratio (aOR) of each group decreased with adding additional confounders (especially risk factors for hemorrhagic stroke). These associations were potentially interacting by other risk factors related to ICH, which was a consistent finding as with prior studies[6, 11, 12, 16, 17]. In further sensitivity analysis by adding NIHSS as a covariate, the relatively reduced sample size might be underpowered to detect the significant difference between the groups.

The concomitant use of antithrombotic therapy was common at the admission in patients with ICH [14, 18]. In our study, 7.7% patients were taking antithrombotic drugs prior to their onset of ICH. Among them, 1.9% patients received oral anticoagulant. The percentage of prior OAs use in this study was lower than a recent cohort study [14]. The underuse of warfarin [19]could be related to the underestimate of the risk of atrial fibrillation[20] and cost of oral non-vitamin K antagonists.

Our study revealed that prior OAs use was associated with a higher in-hospital mortality, which was consistent with former studies [11, 12, 14]. This phenomenon could be due to the increased severity of neurological deficit in our registry. Patients with prior OAs in our study had higher NIHSS and lower GCS scores, as observed in previous studies [11, 12, 14], and had more in-hospital complications (pneumonia, etc.) than no-ATT patients. On the one hand, patients taking oral anticoagulants had specific clinical indications, e.g. atrial fibrillation, which could lead to disparity of baseline characteristics between groups. As in our registry, patients in the OAs group had the highest proportion of AF. On the other hand, OAs were associated with higher baseline hematoma volume and early extension of hematoma [9-11], which were crucial to the worsening of outcomes. However, one of the limitations of our study was that the information on imaging was not available in the database. In recent years, with the widespread use of NOACs, the comparison between warfarin associated ICH (VKA-ICH) and oral non-vitamin K antagonist associated ICH (NOAC-ICH) has become focus of research. NOAC-ICH was reported to have smaller ICH volumes on admission [6, 7, 9, 10], less neurological deficit [7, 9, 10], lower percentage of early hematoma expansion[6],lower in-hospital mortality [6, 14, 21] and conflicting 3-month functional outcomes [10] compared with VKA-ICH. However, since the specific categories of anticoagulation drugs were not recorded, therefore, the comparison between VKA-ICH and NOAC-ICH was not available in our study.

It is important to note that prior combined use of OAs and APT in ICH was surprisingly common and associated with higher in-hospital mortality in our study. The combined preceding use of OAs and APT was not unusual among ICH patients [14, 18]. In patients with atrial fibrillation and also needed coronary vascular interventions, they were on both anticoagulation and antiplatelet therapy for preventing strokes and coronary heart diseases [22].

The association of prior antiplatelet therapy and outcome in patients with ICH is controversial. Previous meta-analysis revealed that APT use at the time of ICH was independently associated with increased hematoma growth [5], mortality [16], but not with poor functional outcome compared with no-ATT use [17]. However, another study provided totally different results [8]. In a large, nationwide ICH registry, the authors reported that dual-ATT rather than single-ATT was associated with higher risk of in-hospital mortality [23]. Another recent nationwide population-based cohort study revealed that prior antiplatelet therapy of phosphodiesterase inhibitor (dipyridamole or cilostazol) was not associated with a poor outcome in patients with ICH[24]. In our registry, a higher in-hospital mortality of prior APT use over non-ATT was not found.

Interestingly, in the subgroup sensitivity analysis, the association between high in-hospital mortality and use of OAs or both OAs and APT was detected in only patients older ≥ 65 years. Older age is an independent risk factor for mortality among ICH patients receiving prior ATT [11] and higher increased risk for ICH among atrial fibrillation patients receiving oral anticoagulation therapy [25, 26]. However, the mechanism regarding the age difference upon preceding ATT use among ICH remained unknown and further investigation is needed. Cautions should be taken when initiating anticoagulation or combined OAs and APT in elder patients.

Our study has several limitations. First, the retrospective analysis from a prospective registry has its inherent selection bias. Therefore, we used several models to adjust for the potential covariables. Second, prior antithrombotic therapy was recorded according to the duration of use. The exact dosages and duration of antithrombotic prior to the development of ICH were not available. Third, the specific categories of antiplatelet or anticoagulation drugs were not recorded in the registry, therefore further analysis regarding comparison between NOAC-ICH and VKA-ICH or single-antiplatelet and dual-antiplatelet therapy would not be available. Fourth, the precise cause and time of death were not recorded and unanalyzed in our study. Fifth, the neuroimaging data of hematoma location, volume and expansion were not available in our database, which could help in defining potential etiology and severity of ICH. We used the baseline NIHSS or GCS scores and risk factor like hypertension as surrogates. Sixth, the preceding antithrombotic information was dependent on the subjective report of patients or families, and some unmeasured confounders or observation bias were not avoidable.

In summary, our study revealed that in patients with ICH, preceding antithrombotic, anticoagulation and combined of anticoagulation and antiplatelet therapies, but not prior antiplatelet therapy alone, were associated with increased odds of in-hospital mortality compared with those received no-antithrombotic therapy, especially in elderly patients. How clinically these antithrombotics can be better used and hemorrhagic complication minimized remain to be studied.
